# Extraction of Antioxidant Phenolic Compounds from Brewer’s Spent Grain: Optimization and Kinetics Modeling

**DOI:** 10.3390/antiox7040045

**Published:** 2018-03-23

**Authors:** Ramiro A. Carciochi, Carlos A. Sologubik, María B. Fernández, Guillermo D. Manrique, Leandro Galván D’Alessandro

**Affiliations:** 1 Faculty of Engineering, Universidad Nacional del Centro de la Provincia de Buenos Aires, Av. del Valle 5737, Olavarría 7400, Argentina; csologubik@gmail.com (C.A.S.); mbfernan@fio.unicen.edu.ar (M.B.F.); gmanrique@fio.unicen.edu.ar (G.D.M.); 2 ISA, Univ. Lille 1, INRA, Univ. Artois, Univ. Littoral Côte d’Opale, EA 7394-ICV-Institut Charles Viollette, 48 Boulevard Vauban, F-59000 Lille, France; leandro.galvan@yncrea.fr; 3 Centro de Investigación en Física e Ingeniería del Centro de la Provincia de Buenos Aires-CIFICEN (UNCPBA-CICPBA-CONICET), Tandil 7000, Argentina

**Keywords:** brewer’s spent grain, extraction, polyphenols, optimization, modeling

## Abstract

The kinetics of polyphenol extraction from brewer’s spent grain (BSG), using a batch system, ultrasound assistance, and microwave assistance and the evolution of antioxidant capacity of these extracts over time, were studied. The main parameters of extraction employed in the batch system were evaluated, and, by applying response surface analysis, the following optimal conditions were obtained: Liquid/solid ratio of 30:1 mL/g at 80 °C, using 72% (*v*/*v*) ethanol:water as the solvent system. Under these optimized conditions, ultrasound assistance demonstrated the highest extraction rate and equilibrium yield, as well as shortest extraction times, followed by microwave assistance. Among the mathematical models used, Patricelli’s model proved the most suitable for describing the extraction kinetics for each method tested, and is therefore able to predict the response values and estimate the extraction rates and potential maximum yields in each case.

## 1. Introduction

Brewer’s spent grain (BSG) is the solid fraction that remains following wort production, which is the first step of the brewing process. BSG is the main by-product from breweries, representing about 20 kg per hectoliter of beer produced [1]. In addition, this source is rich in oligo- and polysaccharides, as well as in polyphenols, which are widely recognized as having antioxidant and antiradical properties [2]. Since it is mainly used as animal feed or fertilizer, BSG can be considered a valuable and underexploited source of bioactive compounds, with potential application in the cosmetic, pharmaceutical and food industries [3].

Extraction is the initial and most important step in the recovery and purification of bioactive compounds from plant materials. Many factors such as solvent composition, extraction temperature and solvent-to-solid ratio, may significantly influence the extraction efficiency, antioxidant activity and phenolic content. Hence, it is necessary to optimize the extraction conditions to improve phenolic recovery and antioxidant activity. The traditional method of optimization (OVAT, one variable at a time) is laborious and time-consuming, since only a single factor at a time is taken into consideration. In this method, the interactions among factors are ignored, hence the chances of obtaining the true optimum conditions are dubious [4]. To overcome this difficulty, a statistical optimization procedure in the form of response surface methodology (RSM) is used.

The conventional solid-liquid extraction method is made in a batch system (BE), however some assistance from emerging technologies, such as ultrasound (UAE) and microwave (MAE), may be conveniently applied during extraction due to their simple, effective and inexpensive natures. In addition, these green methodologies increase extraction yields and decrease extraction times. In order to evaluate the effect of each one of these methods on extraction, a comparison of the kinetics of the processes under the same operational conditions should be performed. Mathematical modeling is a useful tool that facilitates the design, optimization and control of the studied process. In solid–liquid extraction of bioactive substances from plant materials, several theoretical, empirical and semi-empirical models have been successfully employed [5,6,7].

The objective of this work was to study the effects of different extraction methods on the extraction kinetics of phenolic compounds from BSG. The first aim was to optimize the operational extraction conditions of such compounds in a batch system to subsequently evaluate the UAE and MAE processes under optimized conditions. Finally, several mathematical models were tested in order to select one that can be used to accurately describe the extraction process.

## 2. Materials and Methods

### 2.1. Materials

Brewer’s spent grain from pale barley malt (Pilsen type) was supplied by a local microbrewery in Buenos Aires (Argentina). Folin–Ciocalteau reagent, 2,2-diphenyl-1-picrylhydrazyl (DPPH), and gallic acid were supplied by Sigma-Aldrich Chemical Co. (St. Louis, MO, USA). All other chemicals and reagents were of analytical grade. 

### 2.2. Methods

#### 2.2.1. Optimization of Polyphenols Extraction in Batch System

Batch extraction optimization was performed using a central composite face-centered design by varying the following extraction parameters: temperature of extraction (X1; 40, 60 and 80 (°C)), ethanol content (X2; 60, 70 and 80 (%, *v*/*v*)), and liquid/solid (L/S) ratio (X3; 10:1, 20:1 and 30:1 (mL/g)). These conditions and a fixed extraction time (60 min) were selected as a result of preliminary experiments evaluating one factor-at-a-time approach (data not shown). The experimental design conditions are shown in Table 1. Total phenolic compounds (TPC) and DPPH radical scavenging were determined as response variables of the experimental design. For each response, a quadratic equation model was obtained. Regression analysis was conducted, and the response surfaces were plotted using Statgraphics Centurion XVI (version 16.1.18; StatPoint Technologies Inc., Warrenton, VA, USA). The fit of the models to the experimental data was given by the coefficient of determination (R^2^). In addition, each model was validated by calculating the value of the lack of fit test, in which a *p*-value higher than 0.05 indicates that the model was adequate to predict the response values [8]. 

Prior to extraction, BSG was oven-dried at 60 °C for 24 h and subsequently milled and sieved to obtain particle size less than 0.5 mm. Then, according to each run (Table 1), the BSG powder sample (3, 4.5, or 9 g) was suspended in the specific solvent media (60, 70, or 80% ethanol in water (*v*/*v*); 90 mL final volume) and then submitted to different extraction conditions for 60 min in a batch system of 150 mL capacity with magnetic stirring (300 rpm) coupled to an external circulating water bath connected to a thermostat. Afterwards, samples were centrifuged at 10,677 g for 10 min (Sorvall Legend X1, Thermo Scientific, Waltham, MA, USA) and the supernatant was carefully removed for further analyses. 

#### 2.2.2. Ultrasound and Microwave Assisted Extraction Methods

Under optimal conditions found for BE, extraction kinetics were performed for all three tested methods, taking samples at regular time intervals until 120 min, which were then centrifuged under the above mentioned conditions. Sonication in UAE was applied using an ultrasound probe (VCX500, Sonics & Materials Inc., Newtown, CT, USA) operating in continuous mode at a wave amplitude of 50%, frequency of 20 KHz and ultrasound power of 45 W. MAE was performed in an open-system microwave oven (MPD8520 model, Philco, Ushuaia, Argentina) operating in continuous mode at a frequency of 2.45 GHz and 800 W of microwave power. To avoid solvent loss, a reflux system was connected to the extraction flask.

#### 2.2.3. Total Phenolic Content (TPC) and Antioxidant Capacity Determination

TPC assay in extracts was performed using Folin Ciocalteau reagent under the same conditions described in a previous work [9]. Briefly, 0.1 mL extracts were mixed with 0.1 mL of 2 N Folin–Ciocalteau reagent and 0.3 mL of 20% sodium carbonate solution. The volume of the mixture was adjusted to 2 mL with distilled water and incubated in the dark for 2 h. Subsequently, the absorbance of the samples was measured spectrophotometrically at 765 nm. Results were expressed as gallic acid equivalents (GAE) in mg per g of BSG on a dry weight (DW) basis.

Antioxidant capacity was determined by measuring the ability of the extracts to scavenge the free radical 2,2-diphenyl-1-picrylhydrazyl (DPPH). For the reactions, 50 µL of each extract was added to 1950 µL of DPPH solution (60 µM prepared in methanol) and allowed to stand for 30 min in darkness at room temperature before measuring the absorbance at 517 nm. Results were expressed as a percentage of radical scavenging, as previously described by Meneses [10]. 

#### 2.2.4. Modeling of Kinetics Profile and Statistical Analysis

The extraction kinetics curves obtained for TPC and DPPH assays for each of the extraction methods tested (BE, UAE and MAE) were fitted to three equations, with the aim of finding the most appropriate model for the experimental data. These have been successfully applied to model the kinetics of polyphenol extraction and extensively cited in the bibliography, such as Peleg, Page, and Patricelli (Equations (1)–(3), Table 2). The fitting process was carried out using Statgraphics Centurion XVI (version 16.1.18; StatPoint Technologies Inc., Warrenton, VA, USA) by a nonlinear, least squares regression method. The concordance between the experimental data and calculated value was established by the coefficient of determination (*R*^2^), adjusted coefficient of determination (adj *R*^2^), root mean squared error (RMSE), and percentage average absolute relative deviation (%AARD), according to Equations (4)–(7), shown in Table 3. 

## 3. Results and Discussion

### 3.1. Extraction Optimization in Batch System

The effects of three independent variables on the yields of TPC and DPPH radical scavenging values of extracts are shown in Table 1. Phenolic compounds extracted from BSG ranged from 1.59 to 3.57 mg GAE/g, whereas antioxidant activity varied between 1.86 and 11.93% of DPPH radical inhibition. These values are comparable to those reported by other authors [10,11], whose results ranged from 1.26 to 7.13 mg GAE/g DW and 12.02 to 16.91% inhibition, for TPC and DPPH assays, respectively. A value of 20 mg GAE/g DW for TPC assay was reported by Moreira [1] using an optimized microwave-assisted method under alkaline conditions, thus showing an advantage of these novel extraction techniques compared to conventional ones.

For each response, regression equations evaluating the effects of each factor and their interactions were obtained. After subsequent statistical analysis by the ANOVA test (Table 4), the predictive fitted equation models and significant terms (*p* > 0.05) were obtained (Table 5).

The corresponding coefficients of determination (*R*^2^) of the models were 0.9701, and 0.9864 for TPC and antioxidant activity, respectively. These values showed that more than 97.01% of the total variation in the response was explained by the models. Additionally, the very low *p*-values (<0.0001) in each evaluated response indicated the significance of the model terms. In addition, the non-significant value of lack of fit (*p* > 0.05) showed that the models could be used to predict the results. Finally, response surface plots were established using the fitted models in order to determine the optimal levels of the evaluated variables on extraction of phenolic compounds and antioxidant activity.

### 3.2. Effect of Extraction Conditions on TPC Yield

**Figure 1:**
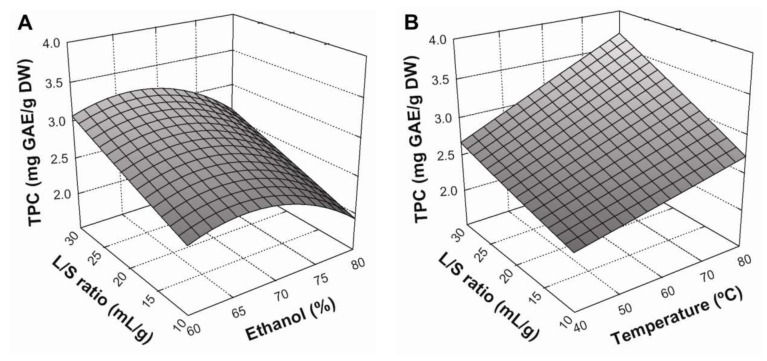

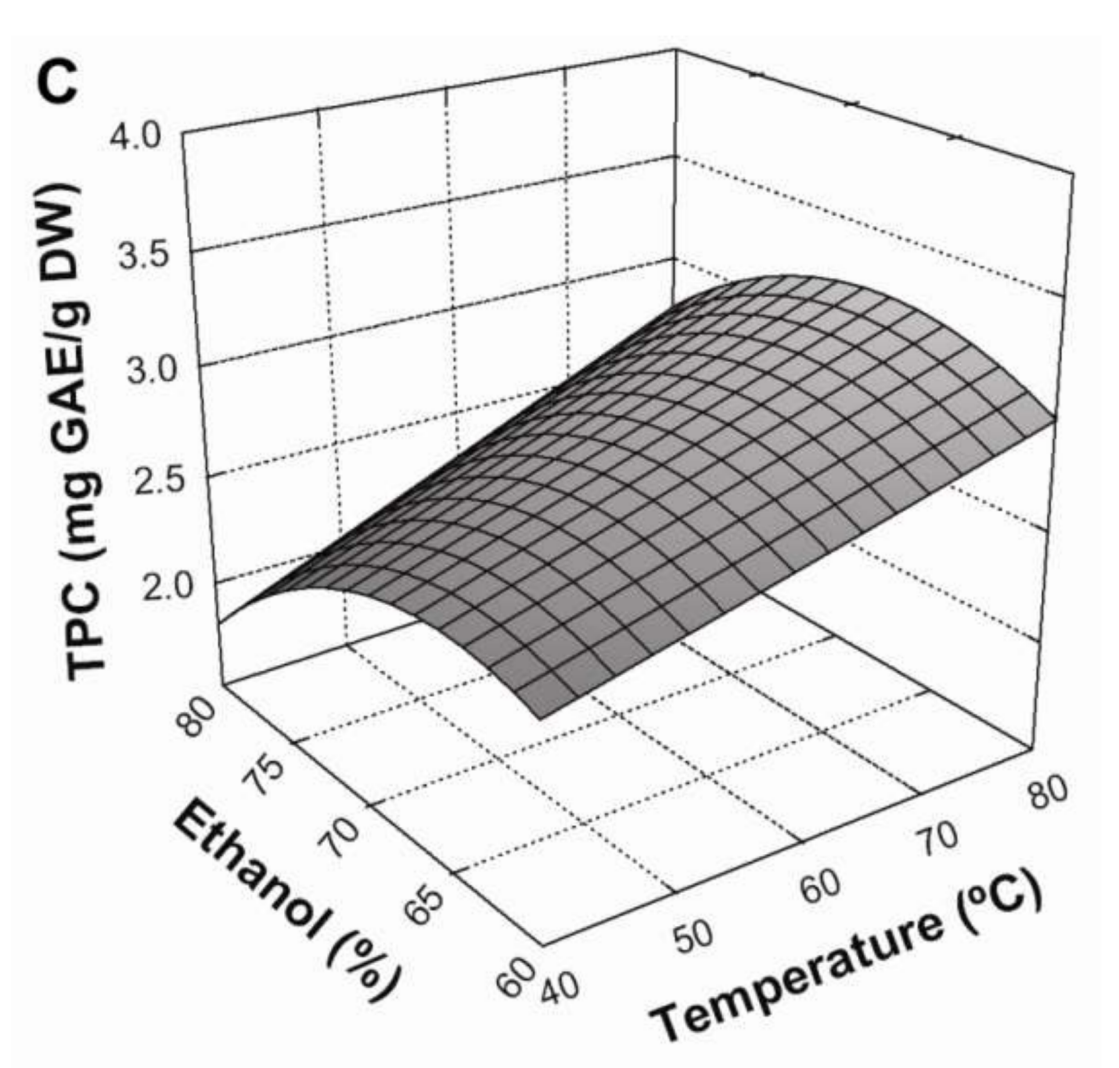
Response surface plots showing the effects of ethanol concentration and liquid/solid ratio (**A**), extraction temperature and liquid/solid ratio (**B**), and extraction temperature and ethanol concentration (**C**) on the extraction yield of TPC in BSG extracts. The missed variable in each graph was kept at the centre point.

Figure 1 presents the response surface plots for the influence of extraction parameters on TPC yield. An increased extraction yield was observed when the liquid/solid (L/S) ratio increased from 10:1 to 30:1 mL/g (Figure 1A). This is concordant with mass transfer principles, since a higher L/S ratio implies higher concentration gradient between the solid and the bulk of the liquid, resulting in a greater driving force for diffusion of compounds to the solvent. This effect was further improved by increasing extraction temperature from 40 to 80 °C (Figure 1B), showing a considerable positive interaction between these variables. It is true that greater temperatures generally improve the solubility and diffusivity of compounds, thus increasing the mass transfer between the plant matrix and bulk solvent [5]. Regarding the effect of solvent composition, TPC yield slightly increased with an increase in ethanol concentration from 60% to about 68%, whilst ethanol concentrations greater than this led to a gradual decrease in TPC yields (Figure 1C). This could be explained by different ethanol:water ratios modifying the polarity of the solvent system, thereby altering the solubility of different phenolic compounds and consequently determining which will be extracted [12]. Thus, the conditions that maximized Equation (8) (Table 5) in a BE process were: a 30:1 mL/g liquid/solid ratio, a temperature of 80 °C, and an ethanol concentration of 66.7% in the solvent system. 

### 3.3. Effect of Extraction Conditions on DPPH Radical Scavenging

**Figure 2:**
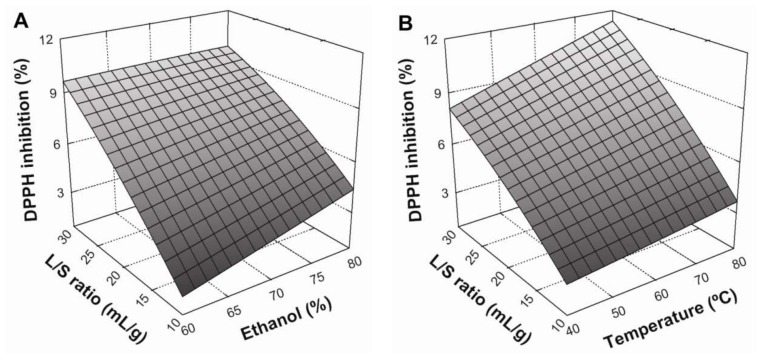

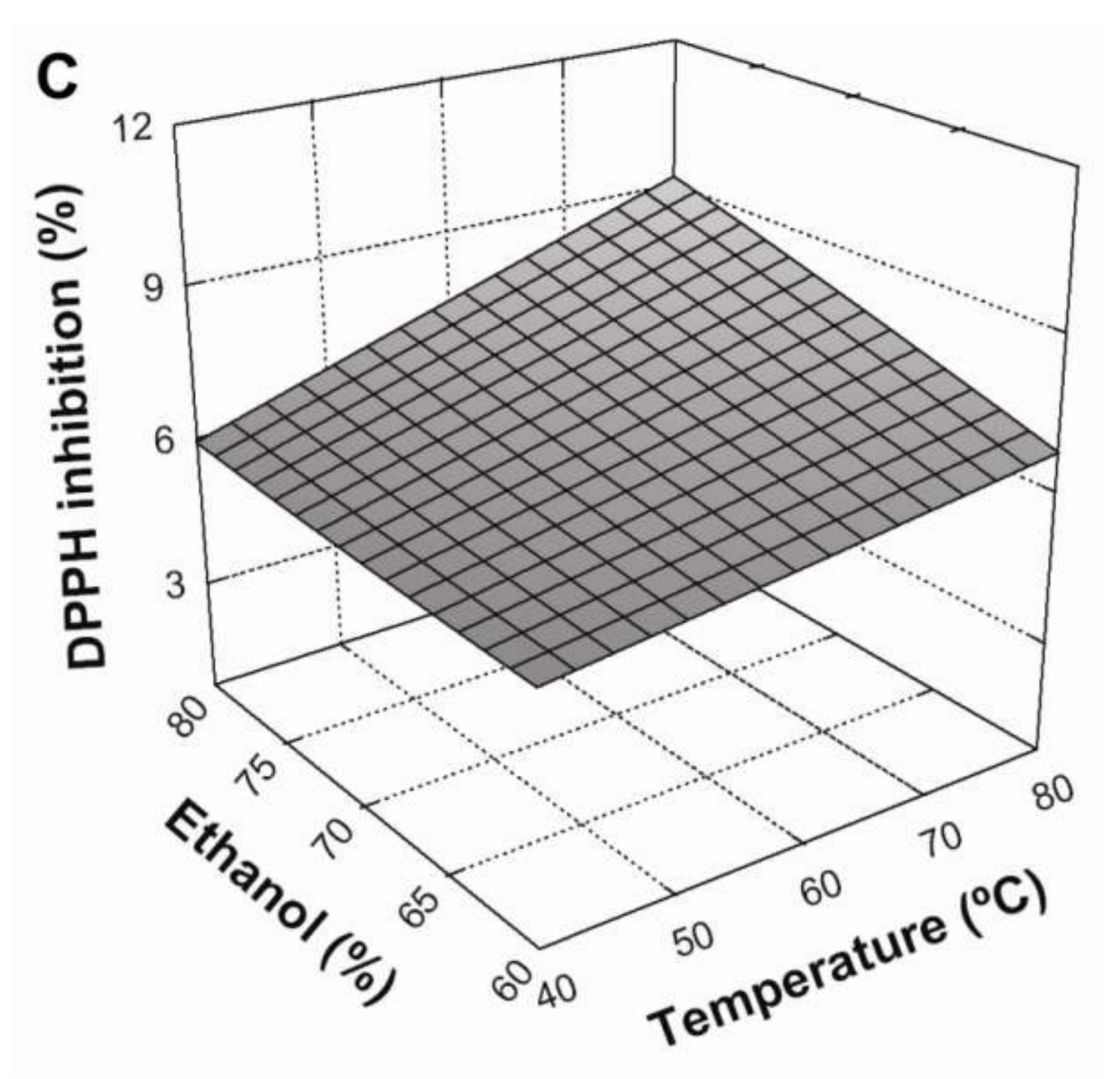
Response surface plots showing the effects of ethanol concentration and liquid/solid ratio (**A**), extraction temperature and liquid/solid ratio (**B**), and extraction temperature and ethanol concentration (**C**) on DPPH inhibition (%) of BSG extracts. The missed variable in each graph was kept at the center point.

According to Equation (9) (Table 5), the liquid/solid ratio was the variable that had the greatest effect (*p* < 0.05) on DPPH radical scavenging of extracts. This was followed by temperature, which had a lesser impact. The influence of extraction parameters on DPPH radical scavenging is shown in Figure 2. Similarly to TPC, DPPH inhibition increased when the L/S ratio increased from 10:1 to 30:1 mL/g and temperature increased from 40 to 80 °C (Figure 2A,B, respectively). Furthermore, the interaction between these variables was significant (*p* < 0.05), with positive effects on DPPH radical scavenging (Figure 2B). Regarding the solvent effect, Figure 2C shows that an increase in ethanol concentration from 60 to 80% produced a positive, linear effect on antioxidant activity. Similarly, the interaction between the solvent system and temperature also produced a positive and statistically-significant (*p* < 0.05) effect. According to these results, maximum DPPH radical scavenging in BE was be achieved under the following conditions: an L/S ratio of 30:1 mL/g at 80 °C, using 80% ethanol as a solvent system.

### 3.4. Optimization of the Extraction Conditions

To optimize the process with two or more output responses, a multiresponse analysis was carried out using the desirability function in the chosen statistical software. The target was to obtain a BSG extract with a high content of phenolic compounds and antioxidant activity. Thus, through maximizing both responses, optimum extraction conditions were obtained: L/S ratio of 30:1 mL/g, 80 °C, and 72% ethanol concentration. Experimental verification of these conditions was performed in quintuplicate, obtaining values of 3.57 ± 0.08 mg GAE/g and 11.55 ± 0.08% DPPH inhibition, for TPC and DPPH assay, respectively. These values confirm the predicted values (3.59 mg GAE/g and 11.55% DPPH radical scavenging) within a 95% confidence level. Hence, under the aforementioned conditions, a BSG extract rich in antioxidant phenolic compounds was obtained.

### 3.5. Extraction Kinetics Study

**Figure 3:**
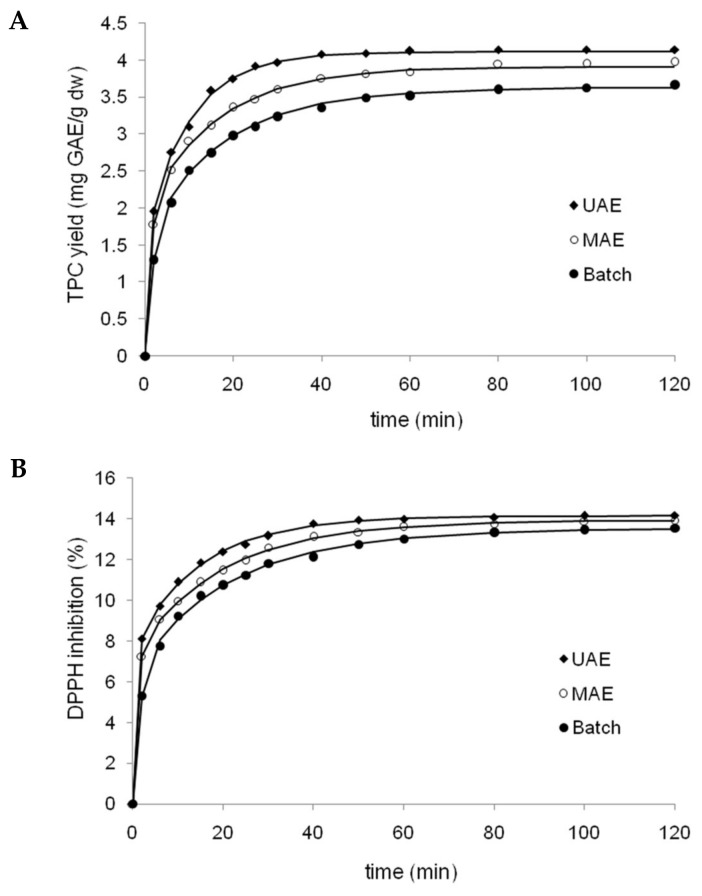
Kinetics profiles of polyphenols extraction (**A**) and antioxidant activity (**B**) of extracts obtained by three tested methods (batch system, ultrasound and microwave assistance) from brewer’s spent grain (BSG) fitted by patricelli’s model. Symbols: Experimental data; lines: Model fitting curves.

Extraction under optimized conditions was performed for a batch system (BE) as well as for assisted processes using ultrasound (UAE) and microwaves (MAE), in order to determine the influence of each extraction method on the yield of phenolic compounds (Figure 3A), and the evolution of extract antioxidant capacity versus time (Figure 3B). Both figures showed a clear, positive effect of assistance techniques, however this was more pronounced for ultrasound assistance than microwave assistance. The mean comparison by Tukey’s test showed significant differences (*p* < 0.05) among the three extraction techniques from 10 to 50 min of extraction for TPC yields. In addition, the kinetics of each extraction method for both responses were fitted to the three equation models presented in Table 2. The corresponding results of nonlinear regression and statistical parameters for BA, UAE and MAE fitted by all three models are shown in Table 6. For both responses, Patricelli’s model was the most accurate fit, with the highest coefficient of determination and adjusted coefficient of determination, and the lowest root mean squared error (RMSE) and percentage average absolute relative deviation (%AARD), compared to Peleg and Page models.

The empirical model proposed by Patricelli [13] involves two simultaneous processes with different kinetics coefficients: a washing stage and a diffusion stage (Equation (3), Table 2). The total amount of extracted solute (equilibrium yield) is equal to the sum of the amounts extracted during both stages. An estimate of the initial extraction rate is given by the first derivative of the equation when *t* = 0 [14]. Thus, according to Patricelli’s model, the equilibrium yields of TPC are 4.11, 3.91, and 3.62 mg GAE/g for UAE, MAE and BE, respectively. These results showed that UAE and MAE significantly increased polyphenol extraction relative to BE, with increases of 13% and 8% for equilibrium conditions, respectively. Additionally, polyphenol extraction was faster using UAE and MAE, according to higher extraction rates (2.42 and 1.66 mg/g/min, respectively) in comparison to BE (0.98 mg/g/min). The observed increase in polyphenol extraction could be due to mechanical effects induced on BSG cell walls, produced during the collapse of the cavitation bubbles (shockwave-induced damage and microjet impact on the surface of the solid material), in relation to MAE where the extraction principle is based on the synergistic combination of heat and mass transfers working from the inside to the outside of the solid sample (in contrast to conventional extraction, in which both transport phenomena occur in different directions) [15,16]. 

According to transfer coefficients calculated for Patricelli’s model (Table 6), the coefficients of the washing stage (k1) for both responses (TPC and DPPH radical scavenging) were much greater than those of the diffusion stage (k2) for all extraction methods tested. As can be seen in Figure 3A,B, all extraction methods showed a quick extraction rate at the outset which was subsequently reduced. This is explained well by Patricelli’s model, since the washing stage allows for quick dissolution of the target components located both at the surface and within broken matrix cells. On the other hand, the diffusion stage is slower due to mass transfer limitations, where the remaining active compounds diffuse from the interior of intact cells into the solvent [5,14]. In addition, a comparison of k values among three methods (Table 6) evidenced greater coefficients for assisted methods relative to BE, mainly in relation to the washing stage. Thus, the assistance of microwaves and especially ultrasound greatly accelerated the washing phase of extraction.

## 4. Conclusions

In this study, optimal extraction conditions for antioxidant polyphenols from BSG in a batch system were obtained using an L/S ratio of 30:1 mL/g, temperature of 80 °C, and 72% ethanol concentration solvent system. Under these experimental conditions, it was clearly shown that ultrasound and, to a lesser extent, microwave assistance increased the extraction rate, equilibrium yield and decreased extraction time. Of the mathematical models describing extraction kinetics tested, Patricelli’s model proved the highest quality fit and was the most suitable for simulating the extraction methods tested. The results of this work could contribute to the optimization and simulation of green extraction process for valorization of agri-food wastes.

## Figures and Tables

**Table 1:** Experimental design of three tested variables with the observed responses values for total phenolic content (TPC) and DPPH radical scavenging.

Run	Temperature (°C; X1)	Ethanol Concentration (%; *v*/*v*; X2)	Liquid/Solid Ratio (mL/g; X3)	TPC (mg GAE/g DW)	DPPH Inhibition (%)
1	40 (−1)	60 (−1)	10:1 (−1)	2.15	1.86
2	80 (+1)	60 (−1)	10:1 (−1)	2.33	2.23
3	40 (−1)	80 (+1)	10:1 (−1)	1.59	3.17
4	80 (+1)	80 (+1)	10:1 (−1)	2.34	5.68
5	40 (−1)	60 (−1)	30:1 (+1)	2.67	8.40
6	80 (+1)	60 (−1)	30:1 (+1)	3.57	10.99
7	40 (−1)	80 (+1)	30:1 (+1)	2.02	7.40
8	80 (+1)	80 (+1)	30:1 (+1)	3.16	11.93
9	40 (−1)	70 (0)	20:1 (0)	2.41	6.50
10	80 (+1)	70 (0)	20:1 (0)	3.19	7.59
11	60 (0)	60 (−1)	20:1 (0)	2.85	5.70
12	60 (0)	80 (+1)	20:1 (0)	2.28	7.85
13	60 (0)	70 (0)	10:1 (−1)	2.59	2.61
14	60 (0)	70 (0)	30:1 (+1)	3.07	9.87
15	60 (0)	70 (0)	20:1 (0)	2.74	7.11
16	60 (0)	70 (0)	20:1 (0)	2.89	6.95
17	60 (0)	70 (0)	20:1 (0)	2.83	6.56
18	60 (0)	70 (0)	20:1 (0)	2.89	6.56

**Table 2:** Equations of mathematical models employed.

Name	Model	Equation
Peleg	$C\left(t\right)=\frac{t}{k_{1}+k_{2}\times t}$	(1)
Page	$C\left(t\right)=\exp\left(-k_{1}\times t^{k_{2}}\right)$	(2)
Patricelli	$C\left(t\right)=C_{1}\left(1-\exp\left(-k_{1}\times t\right)\right)+C_{2}\left(1-\exp\left(-k_{2}\times t\right)\right)$	(3)

*C*(*t*) is the concentration of polyphenols (mg GAE/g DW) at *t* time (min); *k*_1_, and *k*_2_ are constants; *C*_1_ and *C*_2_ are the yields at equilibrium for washing and diffusion steps, respectively.

**Table 3:** Equations of statistical parameters employed.

Statistical Parameters	Equation
$R^{2}=1-\frac{\sum_{i=1}^{n}{\left(Y_{i}-{\hat{Y}}_{i}\right)}^{2}}{\sum_{i=1}^{n}{\left(Y_{i}-\bar{Y}\right)}^{2}}$	(4)
$adjR^{2}=1-\left(1-R^{2}\right)\frac{n-1}{n-m}$	(5)
$RMSE=\sqrt{\frac{\sum_{i=1}^{n}{\left(Y_{i}-{\hat{Y}}_{i}\right)}^{2}}{n-m}}$	(6)
$\%AARD=\frac{100}{n}\overset{n}{\underset{i}{\sum }}\frac{\left|Y_{i}-{\hat{Y}}_{i}\right|}{Y_{i}}$	(7)

*Y_i_* and *Ŷ_i_* are the experimental and calculated values of yield, respectively; *Ȳ* is the arithmetic average value of the experimental points; *n* is the number of the experimental points; *m* is the number of parameters of the regression model.

**Table 4:** Analysis of variance (ANOVA) for the fitted quadratic polynomial model for optimization of extraction parameters.

Source	TPC (*R*^2^ = 0.9701)					DPPH (*R*^2^ = 0.9864)				
	DF	SS	MS	*F*-Value	*p*-Value	DF	SS	MS	*F*-Value	*p*-Value
Model	6	3.83	0.64	59.49	<0.0001	7	134.34	19.19	103.33	<0.0001
Lack of Fit	8	0.10	0.01	2.62	0.2300	7	1.62	0.23	2.90	0.2056
Pure Error	3	0.015	0.005			3	0.239	0.080		

DF, degree of freedom; SS, sum of squares; MS, mean square.

**Table 5:** Predictive model equations of the experimental response variables.

Response	Polynomial Equation	
TPC (mg GAE/g DW)	*y* = 2.83 + 0.38X_1_ − 0.22X_2_ + 0.35X_3_ + 0.10X_1_X_2_ + 0.14X_1_X_3_ − 0.33X_2_^2^	(8)
DPPH radical scavenging (%)	*y* = 6.85 + 1.11X_1_ + 0.69X_2_ + 3.30X_3_ + 0.51X_1_X_2_ + 0.53X_1_X_3_ − 0.60X_2_X_3_ − 0.44X_3_^2^	(9)

**Table 6:** Coefficients and statistical parameters of extraction modelling for all models (*p* < 0.05).

Response Variable	Model	Extraction Method	Coefficient				Statistical Parameter			
			K1	K2	C1	C2	RMSE	*R*^2^	adj*R*^2^	%AARD
TPC	Peleg	BE	1.28	0.27	-	-	0.168	0.939	0.936	2.389
		UAE	0.68	0.23	-	-	0.298	0.825	0.817	7.520
		MAE	0.84	0.25	-	-	0.193	0.919	0.916	3.147
	Page	BE	−0.62	0.17	-	-	0.330	0.796	0.788	9.216
		UAE	−0.90	0.11	-	-	0.310	0.808	0.800	8.120
		MAE	−0.78	0.13	-	-	0.278	0.836	0.829	6.539
	Patricelli	BE	0.53	0.06	1.64	1.98	0.115	0.968	0.967	0.915
		UAE	1.34	0.10	1.63	2.48	0.100	0.954	0.952	0.689
		MAE	0.79	0.06	1.95	1.96	0.105	0.970	0.968	0.770
DPPH	Peleg	BE	0.33	0.07	-	-	0.210	0.993	0.993	3.718
		UAE	0.15	0.07	-	-	0.221	0.988	0.987	4.131
		MAE	0.20	0.07	-	-	0.243	0.988	0.987	5.017
	Page	BE	−1.85	0.08	-	-	0.275	0.988	0.987	6.411
		UAE	−2.13	0.05	-	-	0.226	0.987	0.987	4.323
		MAE	−2.01	0.06	-	-	0.218	0.990	0.989	4.029
	Patricelli	BE	0.61	0.05	6.56	6.93	0.125	0.997	0.996	1.076
		UAE	1.47	0.07	7.72	6.40	0.083	0.997	0.996	0.478
		MAE	1.15	0.05	7.34	6.56	0.070	0.996	0.996	0.342
